# Sound source localization based on residual network and channel attention module

**DOI:** 10.1038/s41598-023-32657-7

**Published:** 2023-04-03

**Authors:** Fucai Hu, Xiaohui Song, Ruhan He, Yongsheng Yu

**Affiliations:** 1 School of Naval Architecture, Ocean and Energy Power Engineering, Wuhan University of Technology, Wuhan, 430063 Hubei China; 2 School of Computer Science and Artificial Intelligence, Wuhan Textile University, Wuhan, 430200 Hubei China; 3 State Key Laboratory of Silicate Materials for Architectures, Wuhan University of Technology, Wuhan, 430070 Hubei China

**Keywords:** Mathematics and computing, Computer science

## Abstract

This paper presents a sound source localization (SSL) model based on residual network and channel attention mechanism. The method takes the combination of log-Mel spectrogram and generalized cross-correlation phase transform (GCC-PHAT) as the input features, and extracts the time–frequency information by using the residual structure and channel attention mechanism, thus obtaining a better localizing performance. The residual blocks are introduced to extract deeper features, which can stack more layers for high-level features and avoid gradient vanishing or exploding at the same time. The attention mechanism is taken into account for the feature extraction stage in the proposed SSL model, which can focus on the most important information on the input features. We use the signals collected by microphone array to explore the performance of the model under different features, and find the most suitable input features of the proposed method. We compare our method with other models on public dataset. Experience results show a quite substantial improvement of sound source localizing performance.

## Introduction

Sound source localization (SSL) refers to estimating the position or direction of arrival (DOA) of the sound source through multi-channel signals. This technology has been well developed over the past few decades and has achieved big progress^1^. Microphone array-based SSL has received a lot of attention from researchers, where DOA estimation is an important research direction in multichannel audio analysis^2^. DOA is usually represented by two relative angles: azimuth and elevation. In most practical cases, SSL is simplified as a DOA estimation problem. Although SSL is a long-standing and extensively researched topic, it remains a challenging problem to date^3^.

Many traditional SSL methods based on signal processing have been proposed, such as steered-response power phase transform (SRP-PHAT)^4^ and generalized cross-correlation phase transform (GCC-PHAT)^5^. Traditional SSL algorithms are based on ideal signal model, so their robustness usually is not very good. With the rapid development of deep learning, more and more researchers are trying to use deep learning methods to solve the SSL problem^6^. Deep learning-based SSL is often formulated as a classification or regression problem. The classification problem divides the space into different regions. For different inputs, the neural network will output its probability values in different regions. The regression problem, on the other hand, estimates the location coordinates or direction of the sound source directly from the inputs^7–10^. With the popularity of deep learning methods, a large number of network architectures and input features are proposed every year, such as convolutional neural network (CNN) and convolutional recurrent neural networks (CRNN), and input features such as short-time Fourier transform (STFT) and generalized cross-correlation (GCC)^11–14^. Many studies have shown that deep learning methods possess good performance^15,16^. Xiao first proposed DOA estimation by neural networks to obtain sound source angle information using a fully connected perceptron^17^. Since then, the SSL technology based on neural network has developed rapidly. Hirvonen uses CNN to extract features from multi-channel amplitude spectrograms, and then classifies the audio source position through four full connection layers^18^. Chabarty proposed a classification method based on CNN to predict the angle of speakers^19^. The input feature is multi-channel short-time Fourier transform phase spectrograms, and the system consists of three continuous convolutional layers and three fully connected layers. Adavanne presented a pioneering work using convolutional recurrent neural networks for SSL and showed good performance^20^. The CRNN consists of multiple convolutional layers and recurrent layers. The convolutional layer has been proved to be suitable for extracting information from various input features. The recurrent layer is suitable for learning time information. Therefore, CRNN is often used for SSL.

In the field of SSL, CRNN is the most popular architecture. However, the existing CRNN-based localization models face a dilemma. It is difficult to extract effective high-level features when the model uses too few convolutional layers, while as the number of convolutional layers deepens, it leads to the problem of gradient vanishing or exploding. Therefore, a sound source localization method based on residual network and channel attention module (SSL-RC) is proposed in this paper. We bring in the residual blocks to extract deeper features, which can stack more layers for high-level features and avoid gradient vanishing or exploding at the same time. Meanwhile, we introduce the attention mechanism into the feature extraction stage of the proposed SSL model, which can focus on the most important information on the input features. As for the input features, we select the original audio captured by the microphone array system as the dataset to train the neural network, and compare and evaluate its localization performance under different input features to find the best input features for our model. Experiments compared with other SOTA SSL models on publicly available datasets show that our model get better performance.

## Related work

The problem of SSL can be analyzed from the perspective of array signal processing, and can also be solved by using the idea of deep learning. Deep learning methods can find the relationship between multi-channel signals and sound source locations. Nowadays the SSL method based on deep learning has gradually become a research hotspot. Generally, the audio signals in different channels received by the microphone array is different, because the distance from the sound source to each microphone are different. Deep neural network (DNN) perform SSL by learning this difference and the complex relationship between input features and sound source location. Many existing researches prove that deep learning methods are feasible for SSL.

Researchers have proposed many neural network-based methods for SSL in recent years. Among these approaches, the majority of model rely on the learning of time–frequency domain features of the acoustic signal. Adavanne implements DOA estimation directly through a set of convolutional layers, a set of bidirectional gated recurrent unit (Bi-GRU) layers and a set of feedforward layers^21^. Lu integrated some additional convolutional layers and replaced the Bi-GRU layers with bidirectional long short-term memory (Bi-LSTM) layers, and this method improved the DOA estimation accuracy^22^. In recent years, more and more researchers have proposed improving methods for classical convolutional recurrent neural networks. Guirguis used temporal convolutional network (TCN) instead of bidirectional recurrent layers, which reduce the computational stress and improve the model inference and training speed^23^. Naranjo improved feature utilization by adding residual squeeze excitation (SE) blocks to the convolutional recurrent neural network. The results show that the introduction of residual SE blocks can obtain better results than the baseline system^24^. Grumiaux proposed a network model with more convolutional layers and pooling layers, which reduced the loss of information and improved the performance^25^. Chakrabarty proposed a CNN based supervised learning method to estimate the DOA of the sound source. The phase component of the STFT of the sound signal was used as the input feature for training. At the same time, the impact of the convolutional layer on the localization performance was evaluated^26^. He used convolutional layers and residual blocks to extract high-level features from the input features for localization, and showed good performance^27^. Komatsu used gated linear units (GLUs) instead of convolutional layers to enhance the learning ability of the frequency dimension, and the model reduces the angular error^28^. Deep learning-based SSL models need to extract useful information from the input features for inference learning, but it contains some information that is not important for the final result. How to make good use of the important information in the features to improve the localization effect of the model is a problem worth studying.

In general, the convolutional module has a direct impact on the performance of the sound source location network model. More and more researches are focused on the optimization of convolutional modules. We will improve the classical convolutional recurrent neural network in this paper. In order to extract high-level features and useful information, we use the residual structure and the channel attention mechanism to improve the information utilization effect.

## The proposed method

We first describe the architecture of SSL-RC, then the specific feature extraction process of the network architecture is described.

**Figure 1:**
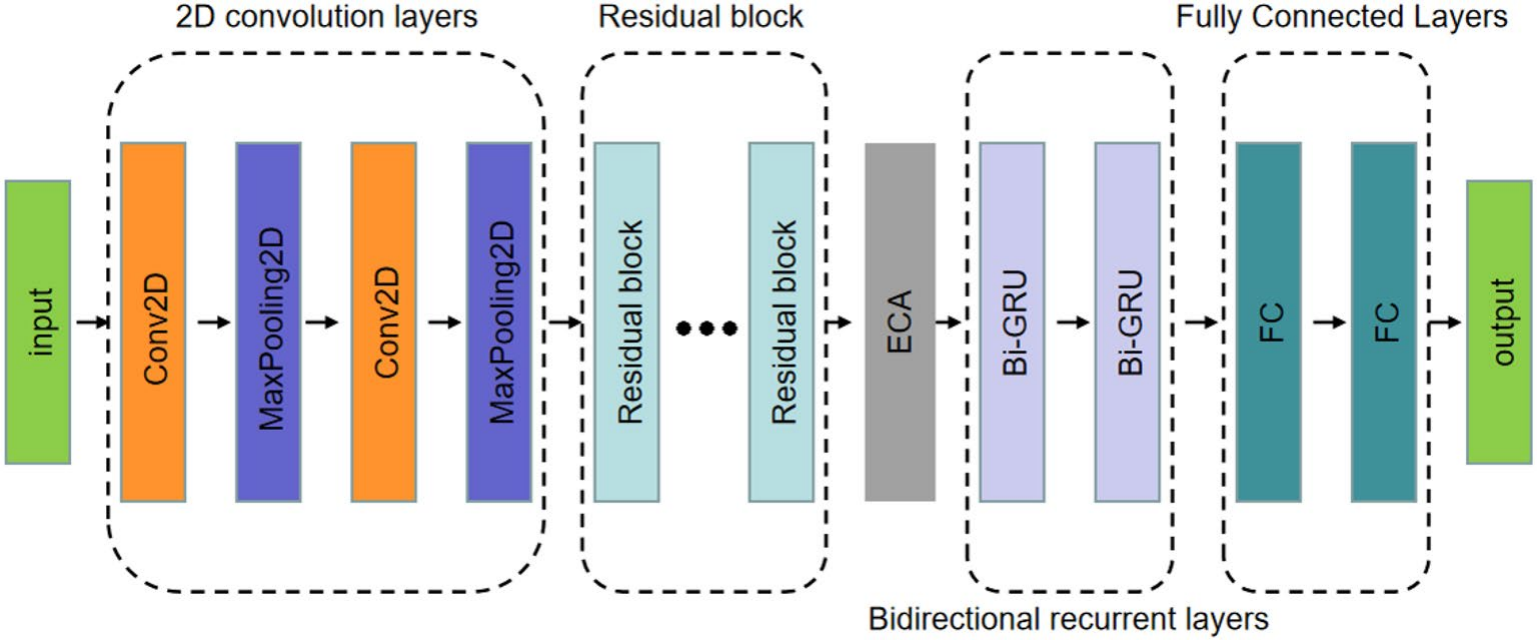
The architecture of the proposed method.

Figure 1 shows the architecture of our model. The input of the model is the feature extracted from the original audio signal. When compared with other models on public data sets, the output of the model is azimuth and elevation angles. When using the data collected by ourselves, the output of the model is probability values of different spatial regions. The main body of the proposed model is mainly composed of residual structure and attention module. The input features are first reduced in frequency dimension size by two two-dimensional convolutional and pooling layers, then five residual blocks are used to complete further feature extraction, after which the attention mechanism is used to achieve channel weight selection for high-level features, and finally the DOA of each frame is obtained by two bidirectional GRU layers and two fully connected layers. Each two-dimensional convolutional layer has 64 convolutional kernels, with a convolutional kernel size of 3 and a step size of 1. At the same time, the batch normalization layer and the max-pooling layer are connected respectively, and ReLu is selected as the activation function. The kernel size of each bidirectional GRU layer is 64 and Tanh is selected as the activation function. The final output is obtained by a fully connected layer. We use Dropout on the standard convolutional layers and recurrent layers to enhance the generalization of the model.

Our model takes the combination of log-Mel spectrogram and GCC-PHAT as the input features. Log-Mel spectrogram is widely used in audio processing. This is found by observing the human ear, which is extremely sensitive to signals in some specific frequency bands. In order to extract log-Mel spectrogram, we first divide the audio signal into overlapping frames, then calculate Fourier transform and apply Mel-scale filter in frequency domain. Finally, the energy in each sub-band is calculated and its logarithm is taken to get the log-Mel spectrogram. GCC-PHAT features are based on the traditional time delay estimation algorithm. For the same sound source, sound waves arrive at two microphones at different times due to sound propagation. Based on the above phenomena, the DOA of sound source can be estimated. GCC-PHAT algorithm uses the correlation between the signals received by two microphones from the same sound source to calculate the cross-correlation function. By maximizing the cross-correlation function, the time delay between the sound source and two microphones can be estimated. It can be seen that the log-Mel spectrogram and GCC-PHAT features of audio signals contain rich time and frequency domain information.

**Figure 2:**
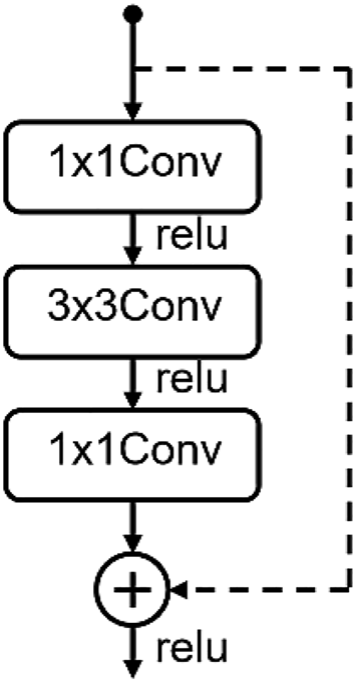
Residual block.

The baseline architecture used for the experiments in this paper is CRNN^20^ which has three convolutional layers followed by two bi-directional GRU layers and two fully connected layers. We note that the traditional convolutional recurrent neural network has too few convolutional layers, which is not enough to extract high-level features, so we add residual layers to enhance the ability of features extraction in time–frequency information. Generally, the more layers the network has, the stronger the expression ability will be. However, more layers may cause gradient vanishing, which degrade the network performance. It is also difficult to avoid these problems by using some regularization and other optimization methods. The residual structure can avoid the problems caused by the increase in the depth of the convolutional layers, so a deeper network can be designed^27^. We choose 2D convolutional layer with residual structure to extract features in our model. As shown in Fig. 2, our residual block uses a three-layer residual unit. The introduction of residual blocks greatly enhances the ability of the model to extract information from input features.

**Figure 3:**
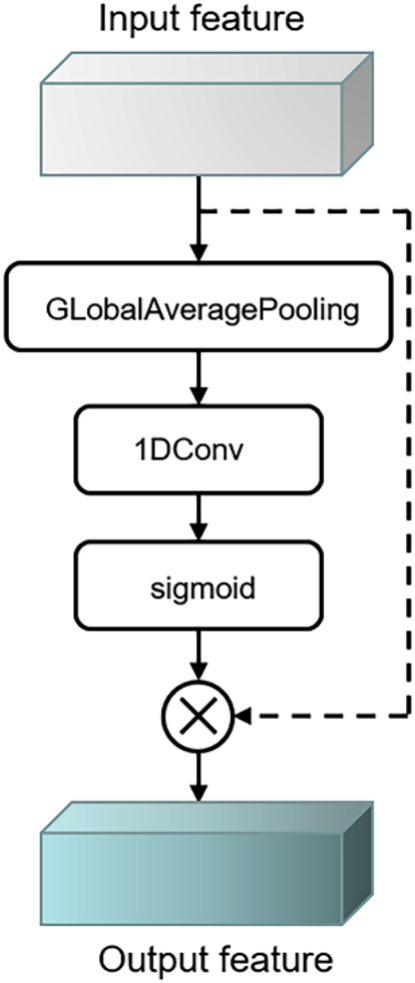
ECA module.

In the traditional convolutional recurrent neural network, the features extracted by the convolutional layer are directly transported to the recurrent layer for time-scale learning, however, the time–frequency features of different channels after convolution may have different influence on the localization performance. We need to learn the weight distribution for different channels, amplify the useful time–frequency features and attenuate the useless time–frequency features. So, we introduce an Efficient Channel Attention (ECA) module before the recurrent layer^29^. It is a local cross-channel interaction strategy without dimensionality reduction, and effectively avoids the effect of dimensionality reduction on the learning effect of channel attention. The module involves only a few parameters but has a significant effect gain, and the proper cross-channel interaction can significantly reduce the complexity of the model while maintaining performance. Considering the outstanding performance of ECA, we introduce this module into the neural network model of SSL. ECA attention mechanism uses one-dimensional convolutional to efficiently realize local cross-channel interaction and extract the dependencies between channels. Firstly, the input features are subjected to global average pooling operation. Secondly, one-dimensional convolutional operation with convolutional kernel size K is performed, and the weight W of each channel is obtained through Sigmoid activation function. Finally, the weight is multiplied by the corresponding element of the original input feature to obtain the final output feature. For important channel, the output of the sigmoid function is close to 1. While for unimportant channel, the output of the sigmoid function is close to 0. Through this module, we carry out channel weighting on the extracted features, so as to efficiently use the information extracted from the residual structure. The ECA module is shown in Fig. 3.

## Experiments

To validate the performance of our proposed network architecture, we set up two experiments. In the first experiment, we compare the proposed method with other model on a public dataset. In the second experiment, in order to find the best features and explore the effect of the model in a real environment, we train the neural network using a real dataset and compare its localization performance using different features.

### Comparisons with other models

We conducted a comparative experiment using a publicly available SSL dataset^7,30^. Convolutional recurrent neural network baseline architecture is very popular in the field of sound source localization. It usually consist of three 2D convolutional layers, two bidirectional LSTM or bidirectional GRU recurrent layers, and fully connected layers^7,20^. We compared SSL-RC with the baseline architecture and other models. At the same time, in order to clarify the influence of channel attention mechanism and the number of residual structures on the performance, the influence of the existence of channel attention mechanism and the number of residual blocks on the localization accuracy and error is compared. The input features used in the model are the combination of log-Mel spectrogram and GCC-PHAT. The number of Fourier transform points is set to 1024, the window length is 1024, the overlap rate is set to 50%, and the window function is a Hanning window. The model is trained using the Adam optimizer. The early stop method is used during training, and the process will be stopped if no improvement in validation loss is observed within 30 epochs. We calculated the percentage of correct prediction angles of the model when the error range is 5°, 10° and 15° respectively, that is, the ratio of the number of correct predictions within the corresponding angle range to the total number of samples in the test set. The mean localization error of the model on the test set was also calculated. The results are shown in Table 1. The best performance in each column is highlighted in bold.

**Table 1:** Ration of correct predictions and Mean localization error comparisons.

Model	< 5°	< 10°	< 15°	Mean
Adavanne^20^	71.11	94.03	97.82	4.29
Tang^7^	69.67	92.09	97.71	4.41
Komatsu^28^	78.3	95.96	98.76	3.64
Grumiaux^25^	75.18	94.48	98.23	3.84
Naranjo^24^	80.83	96.98	99.30	3.14
Guirguis^23^	77.11	96.62	99.01	3.49
CNN	45.96	79.22	90.64	7.43
3Conv-ECA	80.17	96.88	99.01	3.22
3Conv-Res	83.06	97.54	99.07	3.02
SSL-RC	86.53	98.03	99.30	2.73

The results in Table 1 show that the localization accuracy of SSL-RC is improved in different error ranges compared with other models. In the 5° error range, the accuracy is improved by about 5.70–16.86% compared with other models, and our model achieves the smallest localization error. Among the compared models, the CNN performs poorly in the localization metrics due to the lack of learning of temporal information. The 3Conv-ECA model means that we add ECA module after feature extraction block of the baseline architecture. The 3Conv-Res model means that we replace the last convolutional layer of the baseline architecture with five residual blocks. The localization effect of both models has been improved, which also indicates that both residual blocks and ECA module have active influence on the model.

Table 2 shows comparative experiments on the proposed architecture with different number of residual blocks. Models using different numbers of residual blocks with the attention module have higher accuracy than the baseline system. The number of residual blocks also effect the model performance, and the model shows the best performance when the channel attention mechanism is added and the number of residual blocks is 5. The possible reason is that when the number of residual blocks is too small, the model cannot extract enough information, and when the number of residual blocks is too large, the model ignores part of the important information.

**Table 2:** Comparison of model performance with different number of residual blocks.

Config	< 5°	< 10°	< 15°	Mean
3Res	75.38	95.56	98.69	3.74
4Res	83.41	97.38	99.13	2.91
5Res	86.53	98.03	99.30	2.73
6Res	76.73	96.16	98.82	3.52
7Res	75.82	95.19	98.49	3.68
8Res	75.27	94.74	98.27	3.73
9Res	80.73	97.04	99.06	3.27

### Data preparation

**Figure 4:**
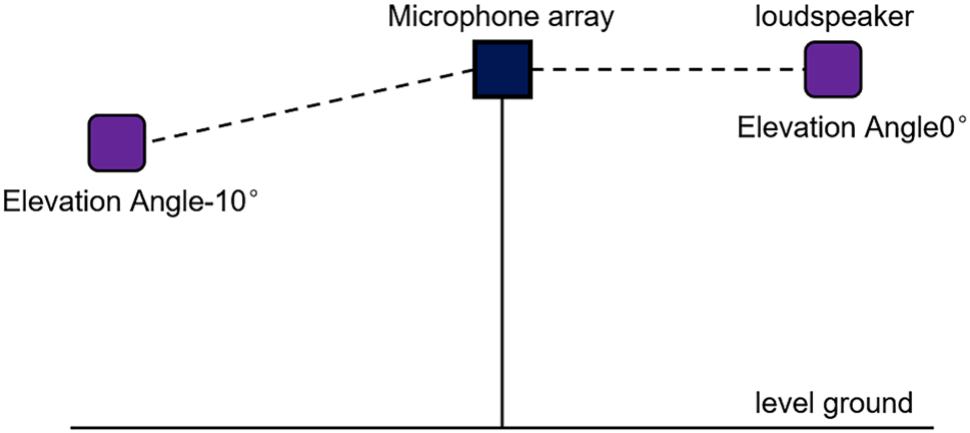
Acquisition of signal.

The input feature selection experiments use audio signals captured by microphone arrays as training data to explore the localization accuracy of the proposed model with multiple input features and find the best-performing features. Deep learning is a data-driven technology, and it usually need a large amount of data to train model. Currently in the field of deep learning SSL, supervised learning is still the dominant approach, which requires a certain amount of labeled data. Most of the current studies use synthetic data to develop neural network models^31,32^. However, synthetic data cannot fully reflect the real environment. We want to use data from real environments for SSL studies. Most SSL algorithms rely on the signal collected by the microphone array. We use a microphone array to acquire audio signals from a real environment, hope to get a dataset for SSL studies. The acquisition process is shown in Fig. 4. The acquisition range of azimuth angle is [− 180°, 180°] and the acquisition range of elevation angle is [− 10°, 10°], using 10° as the division interval, the array element spacing of microphone array is about 0.14 m, and the sampling frequency is 48 kHz.

### Comparison of different features

**Figure 5:**
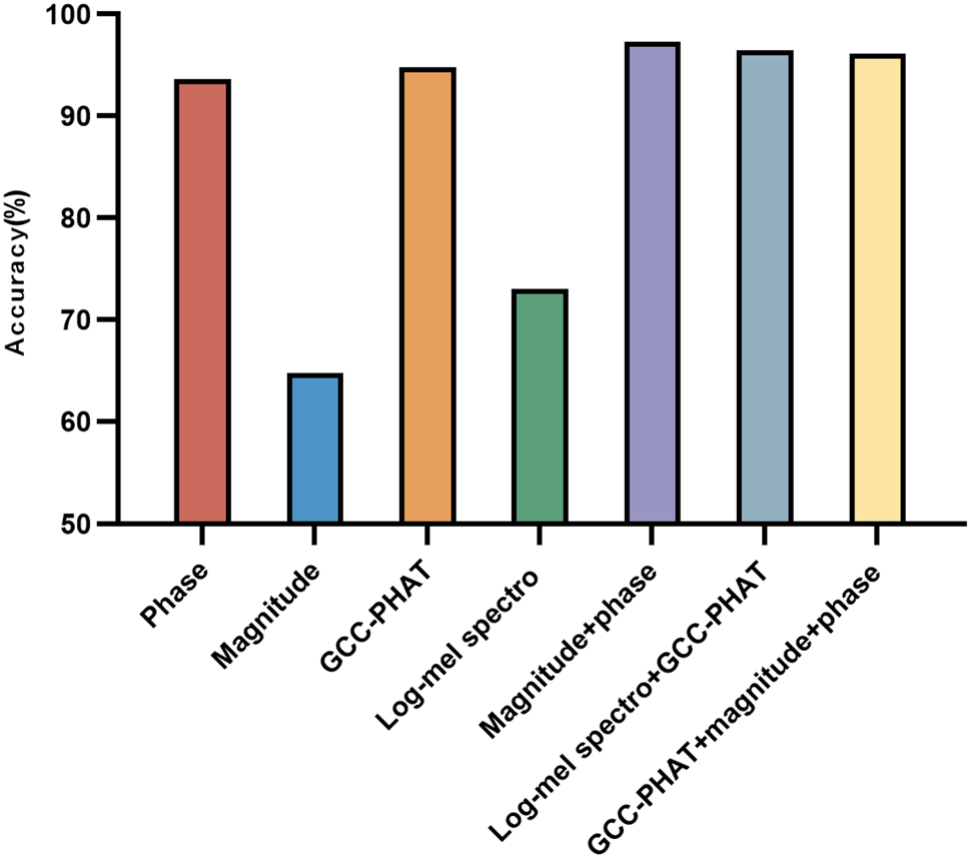
Accuracy of classification of azimuth only.

**Figure 6:**
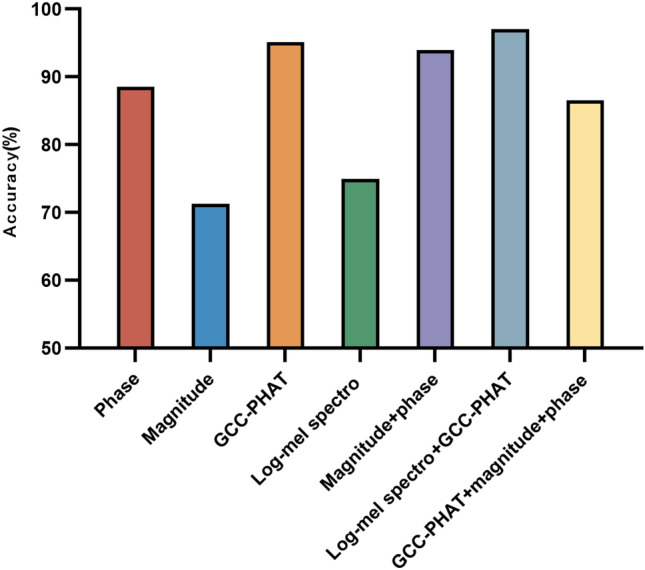
Accuracy of classification of elevation only.

**Figure 7:**
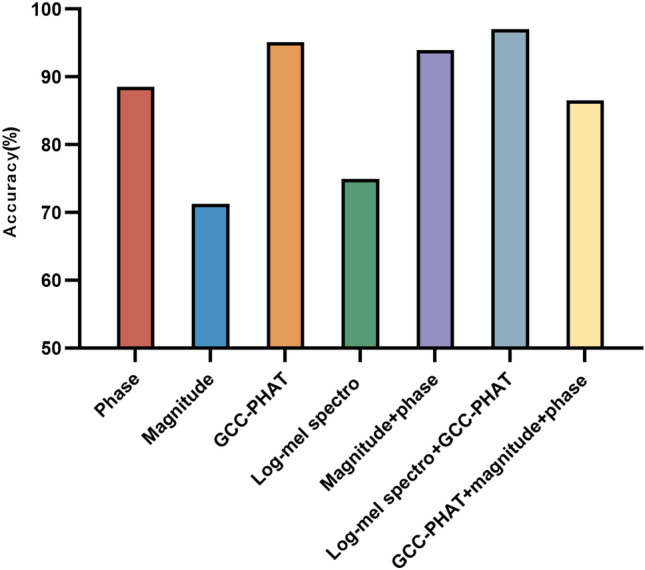
Accuracy of classification of azimuth and elevation.

By synthesizing the analysis of existing literature, we extracted seven input features such as multichannel phase spectrograms, multichannel amplitude spectrograms, log-Mel spectrogram, and GCC-PHAT that are applicable to microphone array audio signals^6^. The Fourier transform parameter settings in feature extraction are consistent with the first part of the experiment. First, we compare the accuracy of each input feature in terms of azimuthal classification, i.e., the ratio of the number of correct classifications to the total number of samples. Figure 5 shows the effect to the azimuthal angle using seven different features. It should be noted that phase represents the short-time Fourier transform phase spectrogram, magnitude represents the short-time Fourier transform magnitude spectrogram, GCC-PHAT represents the generalized cross correlation feature. According to the effect on the test set, the model accuracy using the above seven input features is 93.61%, 64.75%, 94.83%, 73.08%, 97.33%, 96.44%, and 96.12%, respectively. It can be clearly seen that the accuracy of the amplitude spectrogram and the log-Mel spectrogram has a large gap compared with the other features, and their effect is the worst. The accuracy of the other five features is over 90%. The combination of features of the phase and amplitude spectrogram performs almost as well as the combination of features of the log-Mel spectrogram and GCC-PHAT. Figure 6 shows the effect to the elevation angle under seven different features. The overall performance of the seven features is very good, and the accuracy rate is over 95%. At the same time, we also note that the combination of features of log-Mel spectrogram and GCC-PHAT get the best performance. Finally, we compare the effect of the model on joint classification of azimuth and elevation angle. Figure 7 shows the results. The results show that the accuracy of the seven features are 88.52%, 71.30%, 95.15%, 74.94%, 93.94%, 97.01%, 86.58% respectively. The combination of features of log-Mel spectrogram and GCC-PHAT is still superior to other features. They are 1.86–25.71% higher than other features.

## Experimental analysis

To validate the performance of SSL-RC, we first compare it with the baseline architecture and other improved models on a publicly available dataset, and the results show that our model outperforms the other models in terms of localization accuracy and mean error. In the first experiment, the 3Conv-ECA and 3Conv-Res models show the improvement effect in localization accuracy and mean error, demonstrate that residual structure and attention mechanism are helpful to the improvement of SSL performance. In the second experiment, the classification accuracy of the model under different features is investigated. The experimental results show that the combination of features of log-Mel spectrogram and GCC-PHAT outperforms other features, and its average accuracy is 0.76–19.46% higher than the rest of features, as shown in Table 3.

**Table 3:** Average accuracy of the model with different input features.

Input features	Average accuracy (%)
Phase	92.62
Magnitude	78.25
GCC-PHAT	96.44
Log-mel spectro	81.19
Log-mel spectro + GCC-PHAT	97.71
GCC-PHAT + magnitude + phase	92.62
Magnitude + phase	96.95

## Conclusion

This paper presents a SSL model based on residual network and channel attention module. The input features are extracted by the residual network, and then the channels are weighted by the attention module, so that the model can use the time–frequency information more effectively. In order to illustrate the reliability of the proposed model, we compared the proposed model with the popular baseline architecture based on convolutional recurrent neural network and other improved models using the public dataset. Our model shows the best performance in terms of localization accuracy and error. Meanwhile we use the audio signals collected by microphone array in a real environment to study the performance of the model with different input features. The experimental results show that the combination of features of log-Mel spectrogram and GCC-PHAT get the best performance.

## Data Availability

The datasets generated during and/or analysed during the current study are available from the corresponding author on reasonable request.
